# An Epidemiological Study of Filariasis in a Village of District Datia, MP

**DOI:** 10.4103/0970-0218.55284

**Published:** 2009-07

**Authors:** Ashok Mishra, Rahul Singh Bhadoriya

**Affiliations:** Department of Community Medicine, GR Medical College, Gwalior - 474 009, MP, India

**Keywords:** Epidemiolgical study, filariasis, India

## Abstract

**Background:**

District Datia has been known to be endemic for filariasis. A number of cases have been reported in recent past. The present study was an epidemiological investigation carried out in village Barganya, Datia in the month of Sept 2004.

**Aims and Objectives:**

The objectives of this study were to confirm the epidemic in village Barganya and to study the causes of the present epidemic.

**Materials and Methods:**

The study was a cross sectional study carried out through house to house survey, covering a population of 1512.

**Result:**

A total of 78 clinically confirmed cases were reported. The study calculated a microfilarial rate of 6.9% and 1.65% in males and females respectively. The microfilarial density among confirmed cases ranged from 3.1 to 10.6 per 20 cmm.

**Conclusion:**

The study concluded that majority of the cases were males who worked in open field and there was a lack of proper management of these cases at village level.

## Introduction

Filaria is a vector borne disease currently endemic in tropical and sub tropical Africa, Asia, Western Pacific and part of America. South East Asia accounts for about 60 million cases and out of these, India alone accounts for about 40 million cases.(1)

Filariasis is caused by *W. bancrofti, B. malayi and B. timori* and it spreads by the bite of an infected Culex mosquito. *Culex quinquefasciatus* (previously known as *Culex fatigans*) is the main vector for its spread. However, other vectors may also be responsible like Anopheles, Mansonia etc.

The disease has a major socio-economic impact. National Filaria Control Programme (NFCP) was launched in 1955. Initially the programme was limited to urban population but after 1994 the programme was extended to include rural population also. From 2003-04, the programme became a part of National Vector Borne Disease Control Programme (NVBDCP) and it aimed to eliminate lymphatic filariasis by 2015 under National Health Policy 2002.(2)

In Madhya Pradesh, the disease has been endemic in few selected districts. These districts have been included under NFCP and there have been regular distribution of DEC tablets in these districts. However, these strategies do not seem to be effective in achieving the goal of eliminating lymphatic filariasis by 2015 as there have been regular reports of increased incidence of filariasis in these districts. The present study is an epidemic investigation of one such epidemic carried out by Deptt. of Community Medicine, G.R. Medical College, Gwalior in village Barganya, District Datia with the objectives of confirming the present epidemic and to study the cause of present epidemic and the factors associated with it.

## Materials and Methods

There was a suspected epidemic of filariasis in village Barganya, Distt. Datia in September 2004. Deptt. of Health and Family Welfare, Government of Madhya Pradesh requested Dean, G.R. Medical College, Gwalior to carry out an epidemiological investigation in the affected village. A team was constituted with the help of staff, PG students and technicians from Departments of Community Medicine and Surgery to carry out the survey. A study protocol was formulated and it was approved by scientific committee of the college. The team from the Medical College was also assisted by doctors and paramedical staff from Datia.

Barganya is situated approximately 71 km from Gwalior and about 15 km from Datia. The village is easily accessible by road. The nearest Government Hospital is at Sonagiri which is about 4 km from the village. The members of the investigating team divided themselves into small groups comprising of senior staff from Departments of Community Medicine and Surgery, PG student and technician to cover nearly the whole village. The village had a population of about 2201 (Total males 1128: Total females 1073). However, only 1512 villagers turned out for investigation. Rest of the persons refused to participate either due to hesitation or they had no time for investigation. The objectives of the study were told to the villagers and verbal consent was sought from all those who participated in the study. The villagers, who participated, were examined clinically in the day and were informed about the night blood film (NBF) examination. Two slides of night blood film were made, one was examined at District Hospital Datia and the other was examined at Medical College, Gwalior. Clinically confirmed cases were further investigated regarding their socio-demographic profile, environmental conditions, previous treatment taken, if any and the complications associated with it.

## Result

The present study is an epidemiological investigation of a suspected filarial epidemic in village Barganya, Distt. Datia. A total of 1512 villagers participated in this study of which 789 were males and 723 were females. They were screened for any genital swelling at the time of investigation or in the recent past and were subjected for NBF examination. 78 subjects were identified as having filariasis on the basis of clinical examination. NBF examination reported 55 positive cases (43 males and 12 females) of which 12 were those who also showed signs and symptoms of filariasis while rest were asymptomatic.(*P*<0.001 X^2^=28.94 after Yates correction)

The socio-demographic profile of the clinically confirmed cases is given in Table 1. There was a female of age 9 months who showed elephantiasis indicating the recent transmission in the village.

**Table 1:** Socio- demographic profile of confirmed cases

	Male	Female	Total
Age (years)			
<10	1	1*	2
10-20	6	0	6
21-30	16	3	19
31-40	17	4	21
41-50	11	5	16
51-60	7	2	9
61-70	3	1	4
>70	1	0	1
Total	62	16	78
Education			
Illiterate	25	10	35
Literate			
Up to 5^th^	20	4	24
Up to 12^th^	9	2	11
Graduate	8	0	8
Postgraduate	0	0	0
Total	62	16	78
Occupation			
Unemployed/Housewife/Students	8	11	19
Farmers	26	0	26
Unskilled labourers	19	5	24
Businessmen/Shopkeepers	18	0	7
Service (Govt./Pvt.)	2	0	2
Total	62	16	78
Duration of stay			
Since birth	53	3	56
<5 years	0	2	2
5-10 years	0	1	1
>10-15 years	3	2	5
>15 years	6	8	14
Total	62	16	78

The microfilarial rate for males and females was calculated to be 6.9% and 1.65% respectively. The microfilarial density among confirmed cases ranged from 3.1 to 10.6 per 20 cmm of blood. Majority of the cases reside in kachcha houses followed by semi pucca houses [Table 2].

**Table 2:** Distribution of cases according to the type of houses

Type of houses	Number	Percentage
Kachcha	58	74.35
Semi-pucca	17	21.80
Pucca	3	3.85
Others	0	0
Total	78	100

It was observed that majority of the houses where clinically confirmed cases were reported had mosquito breeding places close to them (75.4%). The most common mosquito breeding places noted were ill maintained drains (84.6%), followed by open ditches (26.9%), septic tanks (7.6%), soak pits (5.12%) and others (5.2%). More than one breeding place was noted from most of the houses. Most of the houses where clinically confirmed cases were reported did not have proper system for waste disposal. Only 11.5% of the houses had proper liquid waste disposal facility and 25.6% of houses had solid waste disposal facility. Similarly, only 21.8% of houses had proper sewage disposal system.

It was calculated that only 23.07% of the cases used some form of protection for prevention against mosquito bite [Table 3].

**Table 3:** Protection against mosquito bite (N=78)

Method	Number	Percentage
Net	5	6.41
Repellant	7	8.97
Coil	5	6.41
Any other	1	1.28
Total	18	23.07

**Figure 1:**
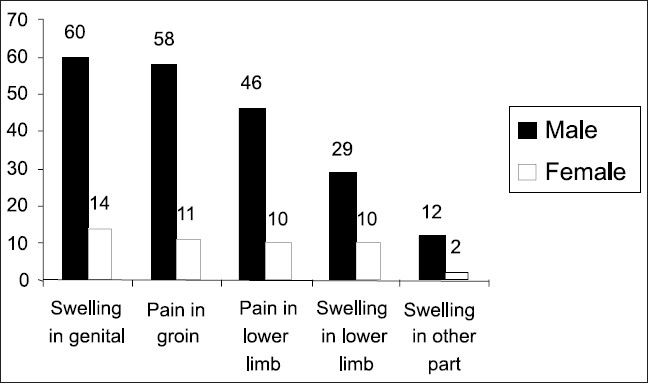
Distribution of complaints among cases

A large number of cases gave no history of any previous injury to genitalia (73.1%) or any similar complaint to other family members (84.7%). Nearly half the number (55.2%) of cases had sought medical or surgical treatment previously from various sources. Nearly all the cases reported having genital swelling or pain in groin. The most common extra genital complication noted was pain in lower limb on walking followed by swelling in lower limb [Figure 1].

## Discussion

The present study has calculated a microfilaria rate (mf rate) of 6.9% among males and 1.65% among females respectively. These rates are comparable to the rates reported earlier in Northern India by various investigators like Patel *et al,* Singh *et al*, Kumar *et al*, Prasad *et al.* and Das *et al.* etc.(3–7) Males have higher microfilaria rate compared to females (X^2^ =14.40 *P*<.001). This can be attributed to the fact that males prefer to sleep in open field and have higher mobility where as females sleep indoor and are not so much mobile.

The study has also calculated microfilaria density in the range of 3.1 to 10.6 per 20cmm of blood which is again comparable to the rates calculated in Northern India by the above mentioned authors.(3–7)

The present study has found that the mf rate was higher among the age group of 31-40 years followed by 21-30 years which has higher economic importance. So any disease that affects this age group is bound to have higher economic impact, as emphasized by the fact that Filaria alone causes an economic loss of Rs. 3500 crores each year world wide.(2) The study has found that majority of the patients affected were farmers or unskilled labourers (64.04%). Other researches had also found similar proportion in their study on rural population.(4,6–8)

The researchers in present study observed that 75.4% of the houses where cases have been reported had mosquito breeding places in their close proximity. This, combined with the fact that 96.15% of cases reside in kachcha or semipucca houses provides maximum opportunity for man-mosquito interaction. A large number of these breeding places were in the form of ill maintained drains (84.61%) and open ditches (26.92%)

It was noted that only a minor fraction of village population pays due attention to proper disposal of liquid and solid waste (11.5 and 25.6% respectively) and only 21.8% had proper sewage disposal system. Negligence on the part of the residents of the village regarding proper disposal of waste and proper sewage system is one of the major causes of endemicity of Filaria in the village. Other investigators who conducted epidemic investigation on filariasis in rural population in various part of country had also made similar observations.(4,6,7)

The study has calculated that only 23.07% of the population used some form of preventive measures for protection against mosquito bite. The most common method used was mosquito repellant while the use of mosquito net at sleeping time was only 6.4%. Patel *et al*. in their studies at Varanasi had calculated this to be higher.(3)

The majority of the cases (55.2%) had sought treatment previously also from various available sources and from various pathies. It indicates the chronicity of the disease and also lack of proper treatment in rural India. The study has also noted that only 26.9% of the cases had history of any trauma to genitals. Only 15.3% had history of similar complaints in other family members. It leads to the conclusion that the disease has no significant association with above mentioned two variables.

The study also observed that nearly all the cases had swelling in genitals (96.7% and 87.6%) and pain in groin (93.5 and 68.7%) among males and females respectively. The most common extra genital complication noted was pain in lower limb on walking, followed by swelling in lower limb. Bhumiratana *et al*.(9) in his study in a village at Thailand and Myanmar border calculated a highly significant association between prevalence of filarasis and hydrocoele. He had also observed that a large fraction of affected population had the most common extra genital complication in the form of pain in lower limb.

## Conclusion

The present study concludes that the majority of the cases reported were among males who work in open field and have high mobility. The chances of acquiring the disease increase if the environment is not clean or proper sanitation practices are not followed. There should be a need to have a proper community based strategy for proper sanitation.

The study also concludes that majority of the cases seek treatment from various sources, but these treatments are insufficient or ineffective. This emphasizes the point that a wider approach is required to involve qualified doctors from different fields for the proper elimination of the disease.
